# CD200:CD200R Interactions and Their Importance in Immunoregulation

**DOI:** 10.3390/ijms22041602

**Published:** 2021-02-05

**Authors:** Katarzyna Kotwica-Mojzych, Barbara Jodłowska-Jędrych, Mariusz Mojzych

**Affiliations:** 1 Department of Histology, Embryology and Cytophysiology, Medical University of Lublin, Radziwiłłowska 11, 20-080 Lublin, Poland; barbara.jodlowska-jedrych@umlub.pl; 2 Department of Chemistry, Siedlce University of Natural Sciences and Humanities, 3 Maja 54, 08-110 Siedlce, Poland; mmojzych@yahoo.com

**Keywords:** CD200, CD200R, cell membrane glycoprotein, immunosuppression, cancer

## Abstract

The molecule CD200, described many years ago as a naturally occurring immunomodulatory agent, capable of regulating inflammation and transplant rejection, has attracted additional interest over the past years with the realization that it may also serve as an important marker for progressive malignancy. A large body of evidence also supports the hypothesis that this molecule can contribute to immunoregulation of, among other diseases, infection, autoimmune disease and allergy. New data have also come to light to characterize the receptors for CD200 (CD200R) and their potential mechanism(s) of action at the biochemical level, as well as the description of a novel natural antagonist of CD200, lacking the NH_2_-terminal region of the full-length molecule. Significant controversies exist concerning the relative importance of CD200 as a ligand for all reported CD200Rs. Nevertheless, some progress has been made in the identification of the structural constraints determining the interaction between CD200 and CD200R, and this information has in turn proved of use in developing novel small molecule agonists/antagonists of the interaction. The review below highlights many of these newer findings, and attempts to place them in the broad context of our understanding of the role of CD200-CD200R interactions in a variety of human diseases.

## 1. Introduction

CD200 is a type-1 cell membrane glycoprotein of the immunoglobulin supergene family, present on both cells with myeloid/lymphoid origin as well as on epithelial cells and many cancer cells [1,2,3,4]. It has been documented over the past decade that interaction of CD200 with its receptor(s), CD200R(s) leads to attenuation of a variety of immune responses, resulting in, as will be discussed in more detail below, prolongation of survival of transplanted allografts [5], as well as, in some instances, decreased resistance to tumor growth. Such observations have heightened the interest in understanding the control of CD200/CD200R expression, their mechanism(s) of action, and the possible development of therapies which might have clinical value in defined scenarios. While additional studies have also documented an important role for CD200-induced modulation in arthritic and other autoimmune diseases [6,7], as well as in allergy [8], infection [9], bone homeostasis [10], and even in spontaneous fetal loss syndromes [11,12,13], the discussion that follows will be predominantly restricted to the consideration of the importance of this molecule as a regulator of transplant survival and tumor immunity.

## 2. Characterization of the Ligands, CD200 and CD200_tr_

### 2.1. CD200

CD200, also known as MRC OX-2, is a highly conserved, 48 kDa [4] type 1a transmembrane glycoprotein related structurally to the B7 family of costimulatory receptors [14], with the gene encoding CD200 located in close proximity to those encoding CD80/CD86 on 3q12-q13 in human (chromosome 16 in mouse) [15,16]. Expression of *CD200* is regulated at the transcriptional level by C/EBP-β [17] which also regulates IFN-γ, IL-6, IL-1 and TNF-α-induced responses [18,19,20]. IFN-γ and TNF-α have been shown to induce CD200 expression in an NF-kappaB, STAT1 and IRF-1 dependent manner [21].

The molecule itself consists of an IgSF extracellular domain (single V + C), a single transmembrane region and a short cytoplasmic tail lacking signaling motifs [1]. The molecule is expressed by resting dendritic cells, thymocytes, endothelial cells, neurons and osteoblast precursors (OBp), as well as by activated B and T cells (including αβTCR^+^ and most γδTCR^+^ cells) [22,23,24]. The glycosylation status of CD200 differs in different tissues expressing the molecule, but to date, there is no evidence suggesting this has significant functional consequences [25].

Examination of mRNA expression for CD200 in various tissues revealed evidence for a splice variant, termed CD200_tr_, derived from exclusion of exon 2 from the full-length form of the molecule. Sequencing suggested this splice variant was associated with a premature stop codon, and it was speculated that no functional protein was associated with its production. This concept was challenged by Chen and Gorczynski, who showed that an artificially created truncated form of CD200 linked to an immunoglobulin Fc region, CD200_tr_Fc, was a natural antagonist of CD200 [26].

### 2.2. CD200tr

By avoiding nonsense-mediated mRNA decay, a splice variant of CD200 which “skips” exon 2, CD200_tr_, can encode a truncated protein following reinitiation of translation at a downstream consensus Kozak sequence [27]. It has been suggested that this protein, lacking ~30 aa from the NH_2_-terminal sequence of the full-length protein, is indeed expressed naturally, and that it acts as the competitive inhibitor to full length CD200. Using in vitro assays, CD200_tr_Fc was shown to antagonize the ability of CD200 to suppress induction of CTL after alloantigen challenge, and to attenuate suppression of IL-2 secretion [26].

Subsequent studies have explored in more detail the expression pattern of CD200 and CD200_tr_. RNA for both molecules is present in all human tissues except skeletal muscle tissues, as well as in B cell lines (Raji, Daudi, TEM). Interestingly, the relative expression pattern of CD200:CD200_tr_ is noticeably altered in human brain and in several neuronal lines (SK-N, HCN-1A) [28]. Furthermore, transfection of cell lines with a vector encoding a genomic construct for CD200 revealed that exogenous expression of CD200 and CD200_tr_ occurred with a similar pattern to that of the endogenous gene in different cells [28]. These observations in turn led to characterization of an exonic splicing enhancer, SF2/ASF:ESE, located in exon 2 of CD200 which controlled the relative levels of CD200/CD200_tr_ expression. Both mutation of the ESE element or blockade of SF2/ASF activity led to decreased CD200 and increased CD200_tr_ expression [28]. The physiological relevance of this was suggested from data supporting a role for altered levels of CD200:CD200_tr_ in animals showing resistance (versus susceptibility) to acute viral infection with a virus causing a SARS-like pulmonary pathology in mice [28].

## 3. Characterization of CD200 Receptors (CD200Rs)

The receptor for CD200, CD200R, is similar in structure to CD200, is located in close proximity to CD200 on the chromosome (in mouse and human) and probably evolved by genetic duplication of CD200. Unlike CD200, more than one isoform of CD200R exists [29], although the best characterized is CD200R1, which is expressed on cells of the monocyte/myeloid lineage and some T cell subsets [5,13,30,31,32]. The majority of human NK cells (CD56^+^, CD3^−^) do not express CD200R1, although in CD56^bright^ human NK cells and NKT cells (CD56^+^, CD3^+^), CD200R is expressed at a low level [33]. The expression of CD200R is significantly up-regulated when human monocytes (CD14^+^, HLA-DR^++^) are induced to differentiate into dendritic cells in vitro in the presence of GM-CSF and IL-4 [23]. While in humans, CD200R1 is presumed to represent the only expressed functional receptor for CD200 [34], in mice, there are data to suggest that both CD200R1 and other alternative isoforms have functional properties following engagement of CD200 (see below). In both human and mice, even mRNA expression of CD200R1 is weak in fibroblasts, endothelial cells and B cells [23].

### 3.1. CD200R1

Human CD200R1 (hCD200R1) is encoded by a gene located at 3q12–13, which spans a region of 52 kb and consists of nine exons. The full ORF contains 1046 nucleotides, and encodes a 348 amino acid protein (CD200R1, isoform 1). Alternate splicing has been suggested to lead to at least three other possible isoforms for hCD200R1 (see alter). CD200R1 shows 53% and 52%, respectively, amino acid sequence identity with rat/mouse CD200R. Like the rodent molecules, hCD200R1 is a plasma membrane protein with a single (V + C) NH2-terminal extracellular domain (aa 25–266), one transmembrane domain consisting of 22 amino acids (aa 267–289) and a short C-terminal intracytoplasmic domain (aa 290–348). The cytoplasmic domain possesses two tyrosine residues located within NPxY motifs (see below), whose phosphorylation is thought to be responsible for triggering an intracellular signaling cascade.

The other three isoforms of hCD200R1 arise from alternative splicing within the sequence encoding isoform 1 and to date have not been described in the rodent counterparts. Thus, a truncated form of CD200R1 (isoform 3) retains the V domain but lacks the C-domain (extracellular), while insertion of an additional 23 amino acids at position 23, encoded by exon 2, generates a isoforms 2 (371 aa) and 4 (211 aa—this form also contains the modification seen in the truncated isoform 3, and possesses only the V domain of the extracellular region). While the presence of the additional 23 amino acids has been postulated to generate a dihydroxyacid dehydratase (DH) domain in hCD200R, the ability of hCD200R to function as a DH enzyme is yet to be confirmed, and its participation in the mitochondrial biosynthesis of valine and isoleucine remains controversial [34].

Most importantly, and as noted above, CD200R1, while closely related structurally to CD200, differs from CD200 by the presence of a long cytoplasmic tail containing two (in human-hCD200R1) [34] or three (mouse/rat CD200R1) conserved tyrosine residues [35]. These critical residues exist at position Y286, Y289 and Y297. Y297 is situated within a conventional NPxY signaling motif [36]. It is worth noting that unlike the majority of other immune inhibitory receptors, CD200R1 does not possess a conventional immunoreceptor tyrosine-based inhibitory motif (ITIM). In an assay which measured the inhibition of mast cell activation in mice, phosphorylation of CD200R1 at Y286 and Y297 was found to be critical for function, with Y289 being dispensable [37]. Upon phosphorylation recruitment of the inhibitory adaptor proteins Dok1 and Dok2 occurred, with resultant inhibition of Ras/MAPK activation [36]. More recent studies by Mihrshahi and Brown have clarified the nature of signaling downstream of CD200R1 engagement [38]. While acknowledging that ligand engagement of CD200R also results in tyrosine phosphorylation of Dok1, this protein was not essential for inhibitory CD200R signaling in human myeloid cells. Indeed, they showed that CD200R-induced phosphorylation of Dok2 preceded phosphorylation of Dok1, and that Dok2 and Dok1 recruited quite different downstream proteins. Thus, while Dok1 recruited less RasGAP than Dok2, Dok2 instead recruited the adaptor molecule Nck following ligand engagement of CD200R. Phosphorylation of Dok1 (following CD200R triggering) resulted in the recruitment of the CT10 sarcoma oncogene cellular homologue-like (CrkL), while the CT10 sarcoma oncogene cellular homologue interacted constitutively with Dok1. Further studies showed that knockdown of Dok1 or CrkL expression in U937 cells caused increased Dok2 phosphorylation and RasGAP recruitment to Dok2. They concluded that a model in which Dok1 negatively regulates Dok2-mediated CD200R signaling through the recruitment of CrkL was the best explanation of the current data.

### 3.2. CD200R2

CD200R2 is one of four alternate CD200R isoforms described for rodent species. Unlike the CD200R1 variant, these alternate receptors are distinct from CD200R1 in that they, like CD200, lack a significant intracytoplasmic signaling domain. However, in contrast to CD200, the transmembrane region of these alternate CD200Rs contains consensus “docking motifs” for adapter molecules (DAP-10/DAP-12), which are then thought to be co-opted into their signaling mechanism(s). Characterization of the alternate CD200R family has been significantly hindered by lack of high-quality isoform-specific reagents [39].

While the binding avidity for CD200R2 and CD200 is significantly less using BioCore measurements with soluble proteins [39], a variety of data suggest that CD200 binding to cell-bound CD200R2 in vivo has functional consequences distinct from those seen following binding to CD200R1, at least in mouse [40]. Indeed, analysis of biochemical signaling pathways activated by binding CD200 to various rodent CD200Rs supports the hypothesis that CD200:CD200R interaction promotes phosphorylation of adaptor DAP-10/DAP12 molecules as part of an alternate (to Dok activation with CD200R1) signaling cascade [23,40,41]. It has been suggested that interactions between CD200:CD200R2 leads to altered development of bone marrow dendritic cell (DC) precursors to produce “tolerogenic” DCs, which in turn promote induction of CD4+CD25+ Foxp3+ Treg cells [40,42].

### 3.3. CD200R3, CD200R4 and CD200R5

CD200R3 and R4 are two other alternate receptors expressed on murine immune cells (basophils, mast cells and bone marrow derived DCs), though to date there is little evidence for a human counterpart [23]. Like CD200R2, both of these receptors could theoretically function as activating receptors, following binding to the adaptor protein DAP12 through a lysine residue located within transmembrane region [43,44].

CD200R3 apparently possesses up to six variant splice forms [23,43], though evidence for their functional expression and/or involvement in immune changes remains to be shown. The full-length CD200R3 consists of 7 exons, in which the first four encode the extracellular region of the molecule (two Ig-like domains). Exon 5, which can be alternatively spliced, encodes the transmembrane domain, and these CD200R3 alternate spliced variants have different affinities for DAP10/DAP12 [43]. Other splice variants can produce soluble (decoy) CD200R3 receptors [43].

In mice, the CD200R4 variant is intriguing because, although it has yet to be shown to have in vivo functional properties, it does have the highest homology with CD200R1, and like CD200R1, high avidity for CD200 [39,41]. CD200R5 may represent a pseudogene which is not expressed [39].

The evidence that CD200 represent a physiological ligand for both CD200R1 and alternate CD200Rs remains controversial (see [23,39] vs. [13,40,41,42,45]). It has been suggested that as-yet uncharacterized low-affinity ligands may represent the natural ligand for alternate CD200Rs [46], with, by inference, the reported interactions of CD200 with alternate CD200Rs representing a non-physiological (or pharmacological) effect.

## 4. Constitutive and Inducible Transcriptional Control of CD200 Expression

Constitutive expression of human CD200 is through transcriptional control of a core promoter region, 169 bp upstream of the major transcriptional initiation site, with the major transcriptional start site 58 bp upstream from the translational start site [17,21]. Within the core promoter region, there exist two positive regulatory domains (PRDs) with binding sites for several known transcription factors. So-called PRD1 contains transcriptional binding sites for Sp1 and C/EPBβ (CCAAT/Enhancer-binding Protein beta), while PRD2 possesses binding sites for Oct-1 and Tst-1. An Ets-1 binding site lies outside of both PRDs. C/EPBβ seems to be the most critical factor affecting both constitutive and inducible expression of CD200 [17,21] and is known to regulate the expression of a number of other genes, including TNFα, G-CSF and IL-8 [47].

Analysis of the 5′-flanking region of CD200 showed the absence of a canonical TATA box [17], while in a region containing cis-regulatory elements probably exhibiting negative functions, there also exist distinct Alu sequences (nt -1249 to -974). However, to date, no disease-associated polymorphisms have been described [17].

Expression of CD200 in T cells, both at the mRNA and protein level, is regulated by TNFα and IFN-γ. Both cytokines induce increased CD200 mRNA expression through a 5′ upstream enhancer. TNFα and IFN-γ promote binding of the following trans-acting factors: NF-κBp65(RelA), STAT1α and IRF-1 and -2 to their cis elements. NF-κB, GAS (Interferon-γ activation site) and ISRE, are all respectively located within a region (~5.4 kB) of a distal CD200 enhancer sequence spanning from -5677 to -5077 nt upstream of the translational start site. A GAS element and NF-κB site are also reported to be present in the CD200 promoter [21].

## 5. Interaction between CD200 and CD200R

**Figure 1:**
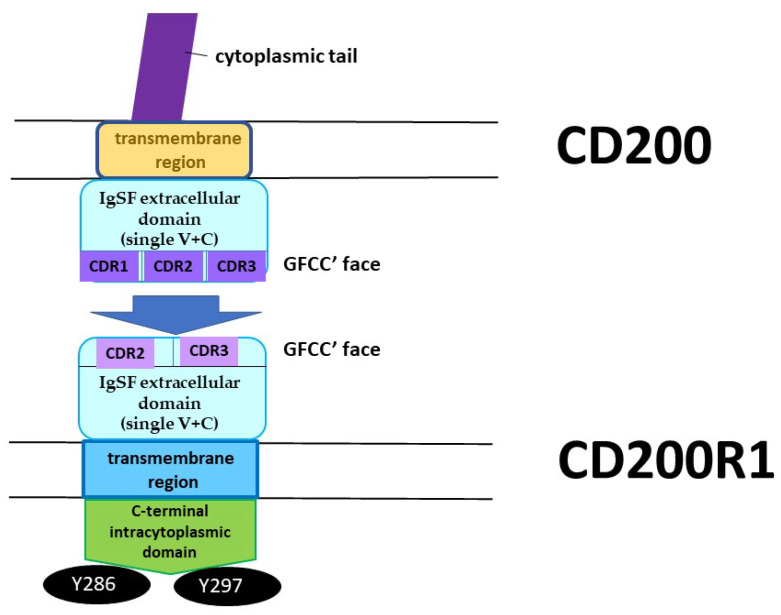
CD200:CD200R1 axis. CD200:CD200R1 interactions occur through Immunoglobulin superfamily (IgSF) extracellular domains, where GFCC′ faces play the crucial part.

CD200 interacts with its receptor isoform(s) through the IgSF domains expressed by both molecules [39], primarily through the GFCC′ faces of their N-terminal immunoglobulin like domains. A key role in this interaction is played by residues E44, I71, T73, E75 and I133, mostly located in the GFCC′ region of CD200R1, although E44 is located outside this region, in the so-called A strand, which is situated within the V-like domain. These conclusions, derived from structural binding data, are consistent with the results of Gorczynski et al., who have used functional studies to characterize in detail the location of the peptide regions that play a crucial role in CD200:CD200R1 interactions both for human and mice. Again, the primary regions of interest were found to lie in N-terminal domains [31,48], with the critical peptides for human CD200R1 located in the F-G loop (CDR3 region: IMVTPDGNFHRGYHL) and the C′-C″ face (CDR2 region: KETNETKET), but not in the CDR1 region (ATNAVLCCPPIALRN). Critical peptides important in human CD200 binding were located in the C-C′-C″, F-G face and even the B face, encompassing all three CDR regions (CDR1: PASLKCSLQNAQ; CDR2: SENHGVVIQ; and CDR3: LFNTFGFGKISGT) [48]. Interestingly, this same group speculated that the epitopes of CD200 expressed within different tissues/cells might not be the same (Figure 1) [49].

## 6. CD200 and Activation of Distinct Intracellular Signaling Pathways

Interaction of CD200 with (one of) its receptor(s), CD200R, delivers different signals to immune cells, likely through the activation of different signaling pathways.

### 6.1. The Role of Dok, SHIP and GASRas Proteins in CD200-Mediated Inhibition of Function in Mast Cells and Human Myeloid Cells

**Figure 2:**
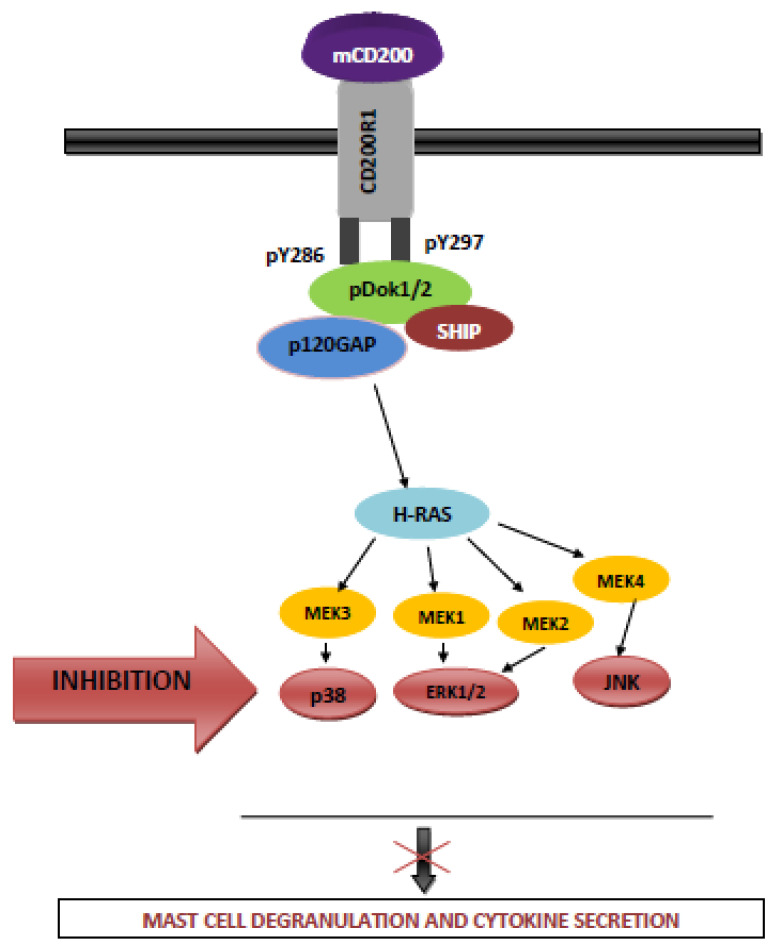
CD200-mediated inhibition of murine mast cells. The same mechanism(s) are operative for CD200R mediated inhibition in human myeloid cells where, in contrast to mice, Dok2 plays a more significant role.

CD200 inhibits murine mast cell activation/degranulation through the inhibition of Ras/MAPK pathways in a process involving Dok 1 and Dok2. Membrane CD200 cross-links CD200R1 and induces tyrosine phosphorylation of CD200R within the NpxY (286/297) motifs [36]. Y297 is a binding site for proteins with a phosphotyrosine-binding domain (PTB), known to be crucial for signal transduction [50], and phosphorylation of Y286 and Y297 of CD200R1 results in the binding of adapter proteins Dok1 (also termed Dok-R) and Dok-2 (also termed FRIP) [36,51,52,53]. This in turn leads to recruitment of RasGAP and SHIP which reduces activation of the MAPKs ERK, p38 MAPK and JNK through the inactivation of Ras (Figure 2) [36].

A similar molecular mechanism has been shown to operate in human myeloid cells (the U937 cell line), although in this case, CD200:CD200R1 interaction recruits Dok2, which in turn through RasGAP activation mediates inhibition—it is thought that any effects of Dok1 are either indirect or absent [38]. This (restricted) role of Dok2 in CD200R-mediated inhibition of human myeloid cell function is apparently dependent on an intracellular tyrosine residue which binds Dok2 with 10-fold higher affinity than Dok1. Dok1 and Dok2 form phosphorylation dependent homo- and hetero-dimers following interaction between their PTB domains and Y146 (Dok1) or Y139 (Dok2) [54,55]. Dok2 now binds with high affinity to tyrosine residues of CD200R1, and phosphorylated Dok2 recruits RasGAP leading to inhibition of function in human myeloid cells. In contrast, Dok1 interacts predominantly with Y917 and Y1022 in the NPxY motif of SHIP [38], although it seems SHIP plays a minor role in any functional inhibition [38].

### 6.2. The Role of Altered Tryptophan Catabolism in CD200-Mediated Inhibition

A potential mechanism for CD200R-mediated suppression of immune system activation was described involving overexpression of indoleamine-2,3-dioxygenase (IDO), which initiates the catabolism of tryptophan and its conversion into *N*-formylo-kynurenin [56]. There are many systems in which IDO overexpression in either DCs or T cells themselves has been reported to produce immune dysregulation [57,58,59]. DCs, particularly plasmacytoid DCs, are primarily involved in IDO-induced tolerance, generally after exposure to IFN-γ and/or TNFα or CTLA4. CD200R ligation also stimulates IDO expression in plasmacytoid DCs, mimicking the effects of B7/CTLA4 signaling, thus reinforcing the tolerogenic properties of some DC subsets over the adjuvant activity of other (immunogenic) subsets [57,60]. Moreover CD200R-triggered signaling up-regulated IDO via type-1 IFN induction, while CTLA-4-induced IDO induction was IFN-γ-dependent [57].

### 6.3. Cooperative Interaction of CD200 and Cytokines in Immune Suppression

**Figure 3:**
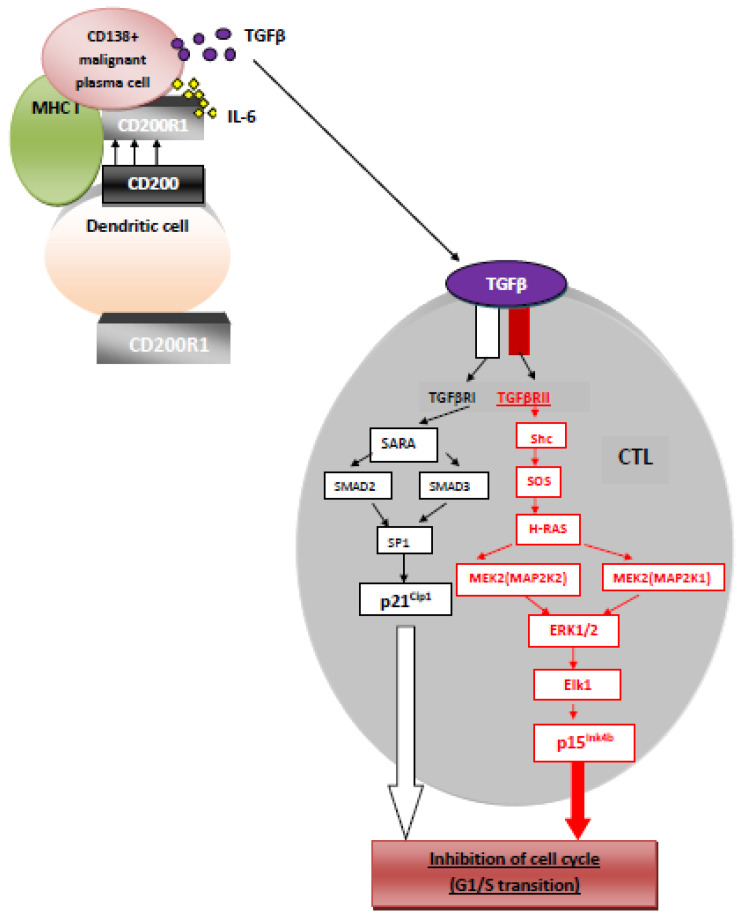
CD200-TGFβRII mediated inhibition.

Since one of the mechanisms by which CD200:CD200R mediated suppression occurs involves downstream augmentation of TGFβ (and IL-10) production [41], a recent study explored the possibility that a bivalent molecule, possessing both a CD200 domain and a TGFβ domain, might represent a more potent immunosuppressant molecule than that predicted from using a combination of these two independent reagents. Indeed, data from this study suggested that this bivalent molecule, linking an APC expressing CD200R with an (effector) T cell expressing a TGFβRII, produced immunosuppression at 10–100 times lower concentrations than the independent sCD200 or TGFβ. Interestingly, unlike suppression mediated by CD200 alone in this model, which seemed to depend upon IDO production (see Section 6.2), the bivalent molecule seemed to produce IDO-independent (though CD200-dependent) suppression [61]. TGFβ-RII kinase is regulated by autophosporylation of, at least, three serine residues: Ser213, Ser409 and Ser416, causing inhibition of CTL activity by cell cycle arrest (G1/S transition) (Figure 3) [62].

## 7. Effect of CD200 and CD200R Expression on Function of Distinct Immune Cells

### 7.1. CD200, CD200R and Dendritic Cells

Expression of CD200 is present on many cell types, including DCs [1], although not all the subtypes of DCs characterized in mice. Expression of CD200Rs is restricted to myeloid-derived APCs and certain populations of T cells [2,22,23].

CD200 expression on DCs increases during apoptosis, an effect explained in terms of p53 response elements (REs) which drive a p53-mediated overexpression of CD200 during DC apoptosis. The p53RE is located within intron 1 for both murine (4 p53REs) and human (2 p53REs) CD200. CD200 expression on DCs is also separately regulated by caspase activity, suggesting the involvement of at least two different pathways influencing CD200 expression during apoptosis. CD200 overexpression during apoptosis alters T cell activity in vitro with the suppression of the production of IFN-γ and TNF-α [57].

Most of these effects are thought to be mediated by CD200R1 engagement. Interestingly, DCs derived from mice lacking CD200R1 and triggered by CD200 are unable to induce CTL in vitro, or graft rejection in vivo [40], but instead preferentially augment activity in, or numbers of, Foxp3+ Treg. It has been hypothesized that such triggering of alternate CD200Rs (particularly CD200R2) on DC precursors leads to this biased differentiation towards so-called “tolerogenic” DCs.

### 7.2. CD200 and Macrophages

Interactions between CD200:CD200R1 in IFN-γ and TNFα activated macrophages (M1-type) derived from human cells leads to inhibition of function through Dok2 and RasGAP. Suppression of macrophage function is implicated in the inhibition of autoagressive T cell responses, inferred from results in CD200^−/−^ mice which show an increased number of CNS-infiltrating macrophages and develop earlier experimental autoimmune encephalitis [31,63]. Elevated expression of CD200R is associated with increased numbers of an alternatively activated (M2a) subtype of human macrophages. These M2a, CD206^+^, cells, generated by IL-4 and IL-13, exert an anti-inflammatory effect and are involved in Th2 immune responses. CD200R- mediated repression of classical macrophage (M1) activation by default allows M2 cells to remain in their polarized state, favoring induction of Th2 immune responses [30].

### 7.3. CD200 and Basophils and NK Cells

Similarly to data reported from macrophages, interaction between CD200:CD200R1 (on basophils/NK cells) results in down-regulation of basophil function (CD123^+^ cells) and also abrogates the lytic function of NK cells [33,64].

### 7.4. CD200 and T cells

Multiple effects on T cells are reported following interactions between CD200:CD200R, including a shift from a Th1 cytokine profile to a Th2 cytokine profile [65], and suppressed CTL responses (Figure 3) [66]. It should be noted that many of these analyses have not eliminated entirely the possibility that regulation of T cell-mediated function by CD200:CD200R occurs indirectly, through, e.g., an effect on CD200R expressed by other cells (DCs, macrophages) implicated in T cell responses. This certainly seems to be the case for the CD200:CD200R2-mediated increase in development of Foxp3 + Treg [42]. Due to the fact that CD200R1 receptor is expressed by some populations of T cells, CD200 may also directly modulate their activity [67]. Rosenblum et al. revealed that co-culture of CD4+ T cells in vitro with autologous DCs from normal CD200+ mice (when compared from CD200-deficient mice) attenuated secretion of pro-inflammatory cytokines (TNFa and IFNg) by autoreactive T cells [68]. Li et al. found that in systemic lupus erythematosus (SLE), the CD200:CD200R axis may regulate CD4+ T population. Namely, in SLE, CD4+ T cells proliferation, which possess decreased (compared to healthy controls) expression of CD200R1, may be downregulated through DOK2 [69].

### 7.5. CD200 Induction by Toll-Like Receptors (TLRs) and NOD-Like Receptors (NLRs)

TLRs and NLRs are pattern recognition receptors (PRRs) that play a pivotal role in the initiation of innate immunity which ensures the first line of protection against invasion of microbial pathogens [70,71]. It has been established that both of these receptors induce CD200 expression. Subsequent CD200:CD200R axis limits the innate immunity, through the inhibition of both classical macrophage activation and other myeloid-derived cells (Figure 2). In this process, the key role plays adapter protein Dok2, that is additionally regulator for TLR4 [72,73]. Existence of such a negative loop feedback seems to significantly protect the host against the development of bacterial sepsis [73,74].

### 7.6. CD200 and MDSCs

Myeloid-derived suppressive cells (MDSCs) that represent a heterogenous population of cells, originating from common myeloid progenitors, are responsible for suppression of immune responses (T cell inhibition, modulation of cytokine production by macrophages and dendritic cells, induction of regulatory T cells) during pathological conditions, such as cancers, infectious diseases, sepsis, bone marrow transplantation, trauma and some autoimmune diseases [75,76]. Like other myeloid-derived cells (mentioned above), MDSCs express CD200R1. Expression of CD200 on tumor cells incites the expansion of MDSCs in tumor microenvironment and increases tumor-increased immunosuppression. CD200 blockade significantly inhibits tumor growth and diminishes the percentage and number of MDSCs that penetrate in tumor tissue. Similarly, sCD200 present in tumor microenvironment of many cancers extends its effects on MDSCs in the same manner [77,78].

## 8. Importance of CD200:CD200R Interactions in Transplantation, Malignancy, Infection and Autoimmune Disorders (Inflammation)

### 8.1. CD200 and Graft Survival

CD200 prolongs allograft survival in a number of animal models (including skin, kidney, cardiac and small intestinal allografts), an effect associated with polarization of cytokine production from lymphoid cells towards increased production of type-2 cytokines (IL-4, IL-10, TGFβ) and decreased production of type-1 cytokines (IL-2, IFN-γ, TNF-α). In vitro, incubation of allostimulated cells in the presence of CD200 leads to inhibition of CTL development, an effect was also seen following in vivo engraftment [5]. It has been established that an immunoadhesin, CD200Fc, in which the extracellular domain of CD200 is linked to an IgGFc region, also induces suppression of alloimmunity in human/mouse cells in culture and supports prolonged allo- and xenograft survival in vivo [5,44]. The mechanism(s) implicated in the inhibition of CTL induction by CD200 involve a role for both IL-10 and TGF-β signaling. Infusion of CD200 (overexpressed on CHO cells) with DCs previously transduced with adenovirus vectors encoding TGFβ or IL-10 also leads to prolonged graft survival [79]. The key role of TGFβ in the prolongation of allograft survival through CD200-mediated effects was further investigated in studies which used administration of the recombinant construct CD200Fc(Gly)_6_TGF-β1, in comparison to CD200Fc or TGF-β alone (or in combination). It was found that the bivalent molecule significantly prolonged skin allograft survival to a greater degree than any other modality used, through a mechanism involving increased Foxp3^+^ Treg (and decreased development of CTL) [61].

Recent studies have attempted to explore the role of CD200 in induction versus maintenance of allograft survival [80]. This report suggests that following establishment of a “tolerant” state in the presence of CD200-overexpression, grafts are maintained by persistent of Treg in the absence of ongoing CD200 expression. However, this persistence is broken in the face of inflammatory stimuli unless CD200 is present. These observations may help explain in part at least the observation that bystander inflammation can often precipitate rejection episodes in previously stable graft recipients.

### 8.2. CD200 and Malignancies

#### 8.2.1. CD200 Expression and Hematological Malignancies

Expression of CD200 as a surface antigen on tumor cells of myeloid/lymphoid origin has been associated with a poor prognosis in a number of human hematological malignancies. Thus, patients with overexpression of CD200 on multiple myeloma cells (MMc), despite high-dose chemotherapy and ASCT, are reported to have a shorter event free-survival (EFS) compared to patients whose MMc do not overexpress CD200 [32].

In studies of chronic lymphocytic leukemia (CLL) interactions between CD200:CD200R were found to be associated with unfavorable prognosis [3,81,82]. Overexpression of CD200 is associated with trisomy +12, which in itself delineates an intermediate risk factor [83]. CD200^+^ tumor cells abolish the ability of PBMCs to eradicate tumor cells [24]. In an in vitro study, blockade of CD200 expression in CLL (by anti-CD200 antibody or siRNAs) decreased the number of CD4^+^CD25^+^Foxp3^+^Treg, enhanced production of the proinflammatory cytokines IFN-γ and TNFα by effector PBMCs, and augmented effective killing of tumor cells by CD8^+^ CTL [81,84]. Following this path, Zhu and colleagues prepared a potentially powerful autologous CLL tumor vaccine. The vaccine preparation assumed purification of CLL cells from peripheral blood mononuclear cells of consenting patients, treating them with an IL-2 and TLR-7 agonist with or without ionomycin, irradiation at a dose of 30 Gy. In the last step, the cells were treated or not with 1B9, which is the rat anti-human CD200 monoclonal antibody. The effect of the tumor vaccine was examined in a human CLL model in NSG mice. CD200 blockade resulted in a decrease of CLL engraftment in peritoneal cavity and increased number of CD8 T cells, which play a key role in tumor B cell killing [85].

The soluble form of CD200, sCD200, in the serum of CLL patients significantly correlates with severity of the disease and may be concern as a prognostic factor for cancer recurrence. Higher levels of sCD200 were found in patients with late stage and/or aggressive disease and with the ones who received two or more courses of treatment [84].

Finally, and consistent with data in MM and CLL, overexpression of CD200 was described in cases of both Hairy Cell leukemia (HCL), ALL and AML and correlated with prognosis for this disease [86,87]. Expression of CD200 at AML, both in secondary and at the diagnosis decreases activity of CD8^+^ T cell cytotoxic potential and the frequency of TNFα-, IL-2- and IFN-γ-producing CD4^+^/CD8^+^ memory T cells, which contributes to the increased risk of relapse and worse overall survival and is independent predictor of cytogenetics [88,89,90]. Moreover, AML CD200 positive patients rarely meet CR after induction therapy [91]. This event may be associated with a significantly increased level of Tregs, which negatively correlates with a response to induction chemotherapy [92].

#### 8.2.2. CD200 Expression and Solid Tumors

Preliminary studies have established expression of CD200 on samples of tumors of various histological grades from lung and prostate tissue and other epithelial-type carcinomas [3]. In a review by Farrar et al., the authors suggested that CD200 expression may be a common characteristic of so-called cancer stem cells, allowing them to evade host immunity [93]. If this is indeed the case, it may be a novel antigen to target for therapy.

In a murine breast cancer model, it was reported that growth of tumor in an immunocompetent environment “selected” for growth of CD200^+^ tumor cells, while expression of mCD200 on breast cancer cells (BTAK^+^) was decreased following growth in immunosuppressed mice [94]. These data were correlated with changes in Foxp3^+^ Treg in the tumor infiltrating population (increased in immunocompetent mice). Once again, levels of a soluble form CD200, sCD200, rose in animals with progressive tumor growth. In preliminary studies, growth of tumor in this model was attenuated using therapy with anti-CD200mAb and was associated with augmented host anti-tumor immunity [94]. Interestingly, use of CD200fc, which is an CD200R1 agonist, significantly decreased the growth of metastatic breast cancer 4THM cells due to the inhibition of cancer-related inflammation and increase both the tumor-infiltrating CD8+ T cell number and tumor-induced IFN-γ secretion [95].

Expression of CD200 in human cell lines derived from melanomas is strongly correlated with transcripts including *MITF*, *endothelin receptor type B* and *silver homolog* [96]. High expression of CD200 is associated with progression from nevi to melanoma and its expression is further increased in melanocytic lesions [97]. The high expression levels of CD200 on human melanomas are again correlated with decreased levels of CTL and a shift from a Th1 to a Th2 cytokine profile, with marked inhibition of IFN-γ [96].

CD200 is also broadly expressed in other solid tumors, such as pancreatic ductal adenocarcinoma, lung cancer, ovarian cancer, renal cell carcinoma, CNS malignancies and head and neck squamous carcinomas (HNSCC), where its interaction with CD200R(1) promotes immune suppression in tumor microenvironment. The immunosuppressive mechanism promoted by CD200:CD200R interaction relies, as mentioned above, on the inhibition of macrophages, induction of regulatory T cells, switching of cytokine profiles from Th1 to Th2, inhibition of tumor-specific T cell immunity and induction of MDSC (myeloid-derived suppressor cells) expansion [77,78,96,98,99].

MDSC are immature myeloid cells, highly elevated in number of tumors, that can suppress antitumor immune responses through the secretion of indoleamine 2,3-dioxygenase, arginase-I, inducible nitric oxide synthase, reactive oxygen species (ROS) and several suppressive cytokines (IL-10, IL-13 and TGF-β) [100,101,102].

CD200 expression is regulated by the *N-RAS/B-RAF/MEK/ERK/MAPK* pathway. The dependence of *ERK* for expression of CD200 helps explain the low levels seen in normal melanocytes and melanoma cell lines where p-ERK is low, and the high levels in cell lines with high expression of p-ERK [103]. It also helps explain the inhibition of expression by the potent *MEK* inhibitor (UO126) and by knockdown of *B-RAF*. Interestingly, while expression of CD200 rapidly decreases using treatment with the MEK inhibitor (UO126), resistance develops to this treatment, consistent with the idea of alternate (*MEK*-independent) pathways of regulation of CD200 expression (transcriptional and/or translational) in human cancer cells.

### 8.3. CD200:CD200R and Their Importance in Infection

It is now known that some viruses express a CD200-like protein, which is thought to contribute to evasion of host immunity following virus infection [9,28,104,105].

The CD200-like protein from the *Herpesviridae* family has the greatest similarity in structure to host CD200 [104]. vCD200 also apparently exists both in membrane and soluble form [106].

Analysis of the genome of the human herpes virus-8/Kaposi’s sarcoma-associated herpesvirus (HHV-8/KSHV), which is associated with the development of Kaposi’s sarcoma [107,108], multicentric Castleman’s disease [109,110] and lymphoma [111,112,113,114,115] in AIDS patients, revealed the existence of an ORF, *K14*, that was homologous to a host CD200 sequence [104]. Although *K14* has only ~40% sequence identity to host CD200, the protein possesses two extracellular IgSF domains (along with a transmembrane region and short cytoplasmic element) and binds host CD200R. HHV-8 K14 protein binding to hCD200R1 has similar kinetics and affinity to that shown by host CD200 ligand (K_D_ ~ 0.5 μM). It is, thus, predicted to deliver regulatory signals to host myeloid cells and affect the severity of viral infection [104].

Since expression of full length CD200 and its splice variant (which has an antagonist role-see above) is controlled by ESE: SF2/ASF interactions, it is of interest to ask whether viral infection alters expression of SF2/ASF and/or the ratio of CD200 to the splice variant natural antagonist, CD200tr [26]. It has recently been claimed that following infection of A/J mice with the coronavirus murine hepatitis virus strain 1 (MHV-1), responsible for the development of an acute SARS-like disease in susceptible mice (severe acute respiratory syndrome), caused an increase in SF2/ASF and full length CD200 (at the expense of CD200tr) and may be associated with in the increased susceptibility of A/J mice (over the resistant BL/6 counterpart) to disease [28].

The role of CD200 expression in chronic viral infection has also been investigated in the influenza model, in which a host response is associated with increased Th1 cytokines and augmented activity in a macrophage (M1) population [116,117]. Airway macrophages have a higher expression of CD200R1 than did their systemic counterparts, thus apparently avoiding the inflammation that typically results from infection. CD200 expressed by the airway epithelium thus represses lung macrophage proliferation and inflammatory lung disease [118]. A functional lack of CD200 results in pathology with influenza-specific NP_366–374_ CD8^+^ T cell infiltration and other pathologies in which CD4^+^ T cells also play a role [119]. Pathology is attenuated by depletion of both CD4^+^ and CD8^+^, although a delay in influenza clearance ensues [120]. These studies emphasize the complexity of the regulation involved in viral immunity (both acute and chronic infection), and of CD200:CD200R interactions in that regulation, and serve as a caution to our attempts to alter the disease outcome in the absence of a more thorough understanding of the normal disease history.

In order to survive in a host, many bacterial and parasitic pathogens adopted the CD200:CD200R axis by modulating the expression of either CD200 or CD200R1, or by expression of CD200 mimic to involve the host CD200R1, which in turn attenuates the innate immunity. This matter was comprehensive described by Vaine and Soberman, who highlighted that both bacterial and parasite organisms through CD200:CD200R interactions (mechanism presented in Section 7.5) may decrease both the release of proinflammatory cytokines by innate cells and their population, and thus, diminish the severity of the disease. For example, *Leishmania amazonensis* induce the expression of CD200 both at mRNA and protein level in bone marrow macrophages, which next inhibit neighboring macrophages that express CD200R1, and thus, abrogate NO production during the infection [74]. In the process of CD200 induction, TLRs or parasite DNA are also involved. Again, Leishmania amazonensis DNA activates TLR9, which in turn induces CD200 in parasite infected macrophages [73]. Infection of helminths (*Taenia crassiceps or Trypanosoma brucei brucei*) leads to overexpression of CD200R on macrophages M2, which in turn inhibits an innate immunity. Namely, M2 cells are embedded in polarized state and enable to create the environment that promote Th2 immune responses [30]. Moreover, exposure to helminth-derived antigens leads to T cell anergy [121].

### 8.4. CD200:CD200R and Their Importance in Autoimmune Disease and Inflammation

Collagen-induced arthritis (CIA) has long been used as an animal model of rheumatoid arthritis (RA). CIA is induced in mice or rats by immunization with autologous or heterologous type II collagen in complete Freund’s adjuvant [122,123]. Susceptibility to collagen-induced arthritis is associated with major histocompatibility complex class II genes, with disease development accompanied by robust T- and B-cell response to type II collagen.

As in RA, pro-inflammatory cytokines, including IFN-γ, TNFα and IL-1β, are abundantly expressed in the arthritic joints of mice with CIA [122,123]. Despite the fact that these Th1-associated cytokines play a pivotal role in the development of CIA, a strong humoral component and the production of type 2 cytokines is also observed throughout the course of disease, suggesting that both types of Th cell responses are involved in modulating arthritis. Administration of a solubilized form of the CD200 molecule, CD200Fc, to mice prevented the development of disease through significant inhibition of sensitization to collagen, with decreased TNFα and IFN-γ in serum, and a decreased release of the same cytokines from collagen-sensitized cells in vitro [7]. An anti-CD200R1 also attenuated existing disease [37].

A role for CD200 expression in endogenous regulation of retinal inflammation has also been investigated. CD200 expression controls myeloid activity, and loss of CD200 expression in CD200 “knockout mice” is associated with activation of M1-macrophages expressing NOS2 (nitric oxide synthase 2), activated microglia and increased Th2 activity [124]. The latter was explained in terms of up-regulation of STAT6 (in spleens and cervical lymph nodes of CD200^−/−^ compared to their wild-type counterparts) which is known to play a key role in polarization towards the Th2 pathway. Nevertheless, these Th2 responses are not thought to contribute to the pathology seen [125]. In independent studies, Dick et al. (2003) have also provided convincing evidence for the role for CD200-mediated immunoregulation in a murine autoimmune uveoretinitis model [6], while Rosenblum et al. (2006) have argued that CD200 expression is intimately involved in controlling inflammation at the “first natural defense barrier”: the skin [68]. He found that CD200:CD200R interactions prevent the initiation of hair follicle (HF)-associated inflammation and diminished all its course by direct quiescence leukocytes naturally resident in the skin. Namely, CD200+ keratinocytes (KCs) located in the outer root sheath (ORF) of murine hair follicles (HFs) act with CD200R+ (positive) leukocytes making them inactivate, which in turn abrogate chemokine-mediated recruitment of other CD200R+ inflammatory cells (macrophage, mast cell) and T cells (also HF-specific autoreactive T cells). Moreover, in murine autoimmune uveoretinitis model, Dick proposed that CD200:CD200R axis indirectly attenuate existing inflammation during autoimmune uveoretinitis model. Briefly, CD200+ endothelial cells and neurons bind to CD200R+ resident macrophages and prevent their classical activation, which in turn mutes the Th1 cytokine response. It is worth to know that these macrophages retain their migratory and phagocytic ability to apoptotic bodies (photoreceptors) and debris.

## 9. Conclusions and Future Perspectives

Since the discovery of its functional role in immunoregulation, a wealth of data have been revealed in both human and animal experimental systems concerning the structure–function relationships for CD200 and its family of receptors, CD200Rs. These data point to an important regulatory role for ligand-receptor interactions in control of, among others, autoimmunity, infection, allergy, transplantation and cancer. Positive and negative effects of CD200:CD200R interaction in various diseases are present in Table 1. More recently, researchers and clinicians have begun to explore the possibility that new therapeutics, based on targeting these interactions, may prove of value in human disease. The biochemical pathways triggered by CD200:CD200R1 interactions are now well-described, but the downstream effects of activating those pathways in different CD200R+ cells are not yet clear. Next, though it has been established that pathogens use TLRs to induce CD200R1 expression, it is suspected that other mechanisms may also be involved. Despite the promising results using an autologous CLL tumor vaccine with rat anti-human CD200 monoclonal antibody in animal model the results of phase I study of samalizumab, which is a recombinant, humanized monoclonal antibody (mAb) that specifically binds to CD200 and blocks its interaction with CD200 receptor (CD200R), in chronic lymphocytic leukemia and multiple myeloma, it did not meet expectations [126]. This is not surprising, as CD200:CD200R, alongside with the other immunosuppressive duo that is PD-L1:PD-1, acts as a tandem to suppress CD8+ T cell function in AML [127]. Therefore, in the future, there is a need to search for alternative pathways/proteins that cooperate with the CD200:CD200R axis. Moreover, regarding the fact that CD200:CD200R1 interactions significantly limit severe autoimmune disorders (see Table 1) it would be reasonable if CD200:CD200R1 modulation can change their paradigm of treatment. Undoubtedly, the best characterized so far are CD200, its receptor CD200R1 and their common interactions. Regarding this matter, it is a challenge to complete the fragmentary knowledge about the importance and significance of the CD200 alternative variant, CD200tr and other CD200 receptors, CD200R2, CD200R3, CD200R4 and CD200R5. We can anticipate that the next decade will see significant advances made in further clarification of these mechanism(s) and in testing hypotheses based on the studies reviewed herein.

## Figures and Tables

**Table 1:** Advantages and disadvantages of CD200:CD200R interactions in multiple human diseases.

Positive Effects of CD200:CD200R Interactions	Negative Effects of CD200:CD200R Interactions
Protects against the development of neurodegenerative disorders (e.g., Alzheimer disease, Parkinson disease) [128,129]	Supports the spread of viral, bacterial, parasite and helminth infections caused by certain viruses (e.g., HHV-8/KSHV, influenza) (see above)
Decreases the development of autoimmune disorders (e.g., CIA, RA, inflammatory retinal diseases etc.) (see above)	Promotes the growth of tumor cells expressing CD200 (e.g., melanoma, breast cancer, prostate cancer, lung cancer, multiple myeloma, acute myeloid leukemia etc.) (see above)
Prolong the survival of allografts, what could be a useful tool in transplantology (see above)	
Reduces the risk of bacterial sepsis [72]	
Promotes recovery after ischemic stroke [130]	
Limits the activity of microglia, and thus, the extent of inflammation in the central nervous system (CNS) (e.g., spinal cord injury) [131]	

## Abbreviations

C/EBP-β	CCAAT/Enhancer Binding Protein Beta
IFN-γ	Interferon gamma
IL-6	Interleukin-6
TNF-α	Tumor Necrosis Factor alpha
NF-kappaB	Nuclear Factor kappa-light-chain-enhancer of activated B cells
STAT1	Signal transducer and activator of transcription 1
IRF-1	Interferon Regulatory Factor 1
SF2/ASF	Pre-mRNA-Splicing Factor 2/Alternative Splicing Factor
ESE	Exonic Splicing Enhancer
NK cells	Natural Killer cells
NKT cells	Natural Killer T cells
GM-CSF	Granulocyte-macrophage colony-stimulating factor
IL-4	Interleukin-4
GAS	IFN-γ-activation site
NPxY	Asn-Pro-any amino acid-tyrosine motifs
Dok1	Docking Protein 1
Dok2	Docking Protein 2
RasGap	GTPase-activator protein for Ras-like GTPase
STAT1α	Signal transducer and activator of transcription 1 alpha
p120GAP	p120-GTPase-activating protein
ISRE	Interferon-Simulated Response Element
SHIP	SH2-containingInositol 5′-Phosphatase
MEK1	Map/Erk kinase-1
MEK2	Map/Erk kinase-2
MEK3	Map/Erk kinase-3
MEK4	Map/Erk kinase-4
MAPKs	Mitogen Activated Protein Kinases
ERK	Extracellular signal-Regulated Kinase
p38 MAPK	p38 Mitogen-Activated Protein Kinase
JNK	c-Jun N-terminal Kinase
IDO	Indoleamine 2,3-Dioxygenase
DCs	Dendritic cells
TGFβ	Transforming Growth Factor beta
CTLA-4	Cytotoxic T-lymphocyte–associated Antigen 4
APC	Antigen Presenting Cell
TGFβ-RI kinase	Transforming growth factor beta receptor I
TGFβ-RII kinase	Transforming growth factor beta receptor II
SARA	Smad Anchor for Receptor Activation
SMAD2	Sma- And Mad-Related Protein 2 (mothers against decapentaplegic homolog 2)
SMAD3	Sma- And Mad-Related Protein 3 ((mothers against decapentaplegic homolog 3)
SP1	Proximal specificity protein 1
SHC	Src homology domain 2 containing
SOS	Son of Sevenless
p21^Cip1^	p21 CDK-interacting protein 1
ERK1/2	Extracellular signal-Regulated Kinase 1/2
Elk1	ETS transcription factor, ETS transcription factor ELK1
MAP2K1	Mitogen-Activated Protein Kinase Kinase 1
p15^ink4b^ alias CDKN2B	Cyclin-Dependent Kinase 4 inhibitor B
p53REs	p 53 Response Elements
CNS-infiltrating macrophages	Central nervous system infiltrating macrophages
Foxp3+ Treg	Forkhead box P3 regulatory T cells
IL-10	Interleukin-10
CHO cells	Chinese Hamster Ovary cells
CTL	Cytotoxic T Lymphocyte
ASCT	Autologous Stem Cell Transplantation
AML	Acute Myeloid Leukemia
ALL	Acute Lymphoblastic Leukemia
STAT6	Signal Transducer And Activator Of Transcription 6
PD-1	Programmed death protein 1
PD-L1	Programmed death-ligand 1
